# Clinical correlates and thyroid hormones of metabolic syndrome in first-episode and drug-naïve major depressive disorder outpatients with and without hyperglycemia: a comprehensive cross-sectional study

**DOI:** 10.1186/s12888-023-05150-8

**Published:** 2023-09-04

**Authors:** Siyang Zheng, Zhiyang Wang, Limin Yang, Xiangyang Zhang

**Affiliations:** 1 College of Basic Medical Sciences, Dalian Medical University, Dalian, 116044 China; 2 School of Medicine, Dalian University, Dalian, 116622 Liaoning China; 3 CAS Key Laboratory of Mental Health, Institute of Psychology, Chinese Academy of Sciences, Beijing, 100101 China

**Keywords:** Major depressive disorder, Hyperglycemia, Metabolic syndrome, Anti-thyroglobulin, Thyroid-stimulating hormone

## Abstract

Hyperglycemia and metabolic syndrome (MetS) are common in patients with major depressive disorder (MDD). This study aimed to explore the prevalence and clinical factors of MetS in first-episode and drug-naïve MDD (FEDND) patients with and without hyperglycemia. A total of 1,718 FEDND patients’ symptoms were assessed using the Hamilton Depression Scale (HAMD), Hamilton Anxiety Scale (HAMA), and positive subscale of the Positive and Negative Syndrome Scale (PANSS). Blood glucose levels, metabolic index, and thyroid hormones were measured during fasting. The prevalence of MetS in FEDND patients with hyperglycemia was 35.67 times higher than in FEDND patients without hyperglycemia. FEDND patients with MetS were older, had later age of onset, and were predominantly married than those without MetS (*p* < 0.05). Among FEDND patients without hyperglycemia, suicide attempts, severe anxiety, HAMD, HAMA, PANSS subscale scores, thyroid stimulating hormone, antithyroglobulin, and total cholesterol levels were all higher in patients with MetS than those without MetS (all *p* < 0.05). In FEDND patients without hyperglycemia, the combination of age and TgAb distinguished those patients with and without MetS. Our results suggest a high prevalence of MetS in FEDND patients with hyperglycemia. Several clinical variables and thyroid function-related hormones impact MetS in patients with FEDND.

## Introduction

Major depressive disorder (MDD) is a major public health problem affecting people of all ages, with negative effects on both patients and society [1]. Patients with MDD suffer psychological effects and are more likely to develop age-related physical illnesses such as cardiovascular disease (CVD), type 2 diabetes (T2D), obesity, dementia, and cancer [2–4]. MDD is conceptualized as a disease that accelerates aging [5]. All parts of the patient’s body are affected by MDD, which alters circadian rhythm, autonomic nervous system function, immune response, and hypothalamic-pituitary-adrenal (HPA) axis activity [6, 7]. MDD increases the risk of death by promoting a decline in physical and cognitive function in patients [8, 9].

The link between affective disorders and abnormal blood glucose was recognized over a century ago [10]. Although the baseline blood glucose levels in patients with MDD are not significantly different from those of the general population, patients with MDD exhibit significant insulin resistance and reduced glucose tolerance [10]. Dysfunction of the HPA axis and disturbances in the norepinephrine and 5-hydroxytryptamine metabolism, observed in MDD patients/animals, have also been found in T2D patients/animals [11–13]. Mendelian randomization studies based on single nucleotide polymorphisms have identified MDD as a potential risk factor for T2D [14]. Patients with MDD are at significantly higher risk of developing T2D than the general population due to sharing the same pathophysiological mechanisms, such as activation of the HPA axis, inflammation and genetic factors [15–17]. Davy *et al*. reported an 8.7% prevalence of T2D in patients with MDD, which is 1.49 times the risk of the general population [18]. Patients with MDD have varying degrees of dysglycaemia, mainly manifested as hyperglycaemia. Our previous study found a high prevalence of hyperglycemia in patients with first-episode and drug-naïve MDD (FEDND) [19]. This may be related to the high prevalence of T2D.

Metabolic syndrome (MetS) is a concept designed to help clinicians identify patients at greater risk for CVD and T2D based on their metabolic profile [20]. It is a preclinical state for the development of T2D and CVD. It describes metabolic abnormalities such as abdominal obesity, hypertriglyceridemia, low high-density lipoprotein (HDL) cholesterol, hypertension, and hyperglycemia [21]. Studies have confirmed a bidirectional association between MDD and MetS, with MDD predicting the onset of MetS and MetS predicting the timing of MDD [22]. Thyroid hormones are major regulators of metabolism, and their receptors are widely distributed in various cells. Small changes in circulating concentrations of thyroid-related hormones will have a significant impact. The role of thyroid dysfunction in exacerbating T2D and CVD has been recognized [24–26]. Subclinical thyroid dysfunction is a biochemical condition that manifests as subclinical hypothyroidism (SHypo) or subclinical hyperthyroidism (SHyper). This condition is characterized by abnormal serum levels of thyrotropin (TSH) and normal serum concentrations of free thyroxine (FT4) and triiodothyronine (FT3). In SHypo, TSH serum levels are above the upper reference limit of normal (> 4.0–5.0 mIU/L), whereas in SHyper, TSH is below the lower reference limit (< 0.3–0.5 mIU/L). SHypo is more common (approximately 3%) than SHyper (0.7%) [23]. In patients with MDD, a high prevalence of SHypo has a negative impact on MDD [24]. SHypo and SHyper, especially SHypo, are associated with an increased risk of CVD and T2D [25–28]. Therefore, thyroid dysfunction is strongly associated with the development of MDD, T2D, and MetS.

All studies on the relationship between MDD and MetS consistently confirm a strong association between MDD and obesity-related components (abdominal obesity, low HDL-C, hypertriglyceridemia), while the association with hyperglycemia is less certain. [29]. Hyperglycemia is closely associated with MetS and is one of the diagnostic indicators of MetS [30]. Based on these findings, we proposed that the prevalence of MetS would be higher in MDD patients with hyperglycemia than in those without hyperglycemia. Clinical factors and factors related to thyroid function would be risk factors for MetS in patients with MDD. Investigating the prevalence and risk factors of MetS in MDD patients with/without hyperglycemia is essential for managing and preventing CVD and T2D in MDD patients.

## Methods

### Study population

The 1718 patients included in this study were from the psychiatric outpatient clinic of the First Hospital of Shanxi Medical University from 2015 to 2017. In this study, only patients who met the following criteria were included: (1) age 16–60 years old, Han nationality; (2) diagnosis of MDD on admission by two trained clinical psychiatrists based on the Structured Clinical Interview for DSM-IV (SCID); (3) never received psychotropic medication or other treatment; and (4) no serious physical illness (e.g., central nervous system disease, and unstable medical conditions).

Patients were excluded if they (1) were unable to provide written informed consent, (2) were pregnant/lactating, (3) had a major physical illness, (4) had a history of any mental disorder other than MDD on Axis I, and (5) had a history of substance abuse or dependence, excluding smoking.

All participants signed an informed consent form, and the study was approved by the First Hospital of Shanxi Medical University (No. 2016-Y27).

### Demographic characteristics and clinical measures

A self-designed questionnaire was distributed to each participant by specially trained research staff. Information was collected through the questionnaire, including age, gender, education, marital status, body mass index (BMI), age of onset, and duration of illness. Information was supplemented by reviewing available medical records and interviewing relatives or primary care physicians.

Depression and anxiety symptoms were assessed using the Chinese versions of the 17-item Hamilton Depression Rating Scale (HAMD) and the 14-item Hamilton Anxiety Rating Scale (HAMA) [31, 32]. MDD patients with HAMA total score above 18 were identified as having anxiety symptoms [33]. The positive subscale of the Positive and Negative Syndrome Scale (PANSS) was used to determine the severity of psychotic symptoms [34]. In the present study, participants with a total score of more than 15 on the PANSS positive subscale were assessed as having psychotic symptoms [35].

Prior to the study, two research psychiatrists were trained in the use of the PANSS, HAMD, and HAMA to ensure consistency and reliability of scoring throughout the study. Their inter-rater correlation coefficient of the HAMD and HAMA total scores was greater than 0.8. These two psychiatrists were blinded to the clinical status of the patients.

### Collection and measurement of blood samples

Fasting serum samples from all participants were collected between 6 and 8 am on the same day as the clinical rating scales. All blood samples were sent to the hospital laboratory for testing immediately before 11 am on the day of collection.

Hormone levels related to thyroid function, including free triiodothyronine (FT3), free thyroxine (FT4), thyroid stimulating hormone (TSH), anti-thyroglobulin (TgAb), and thyroid peroxidase antibody (TPOAb), were measured using a Roche C6000 electrochemiluminescent immunoassay analyzer (Roche Diagnostics, Indianapolis, IN, USA). Fasting blood glucose (FBG) was measured using the glucose oxidase method. The enzymatic colorimetric method was used to measure lipid-related indicators, including low-density lipoprotein cholesterol (LDL-C), high-density lipoprotein cholesterol (HDL-C), total cholesterol (TC) and total triglycerides (TG) [36]. FBG > 6.1 mmol/L was identified as hyperglycemia [37].

In addition, the patient’s BMI, diastolic blood pressure (DBP), and systolic blood pressure (SBP) were also measured. MetS was diagnosed if three of the following four items were met: (1) BMI ≥ 25 kg/m^2^; (2) FBG > 6.1 mmol/L or 2-hour postprandial glucose ≥ 7.8 mmol/L and a diagnosis of T2D; (3) SBP/DBP ≥ 140/90 mmHg or a diagnosis of hypertension; (4) TG ≥ 1.7 mmol/L or fasting in men HDL-C < 0.9 mmol/L or < 1.0 mmol/L in women [38]. The number of items meeting the above four items was used as the MetS index of the patients.

### Statistical analysis

The Kolmogorov–Smirnov one-sample test was used to detect normal distribution of all variables. For categorical variables and normally distributed continuous variables, the Chi-square test, and Student’s t-test were used, respectively. For non-normally distributed variables, the Mann-Whitney U-test was used. Bonferroni correction was used for multiple tests. To investigate MetS risk factors in FEDND patients with/without hyperglycemia, univariate analysis was performed between MetS and non-MetS. In logistic regression, factors significantly differed between MetS and non-MetS were included (Condition: Wald). The area under the receiver operating characteristics (AUC ROC) was used to determine the discriminatory ability of underlying parameters to distinguish patients with and without MetS. A consistency statistic between 0.7 and 0.8 was generally considered acceptable.

Analyses were performed using SPSS version 21.0 (IBM, Chicago, IL, USA). *p*-values were set as two-tailed with a significance level of α = 0.05.

## Results

### Prevalence of MetS in FEDND patients with and without hyperglycemia

The proportion of hyperglycemia in FEDND patients was 13.6% (234/1718). HAMA and HAMD scores were higher in FEDND patients with hyperglycemia compared to FEDND patients without hyperglycemia (both *p* < 0.001).

The prevalence of MetS in patients with FEDND was 6.93% (119/1718). The prevalence of MetS was 35.67 times higher in FEDND patients with hyperglycemia (101/234, 43.16%) than in FEDND patients without hyperglycemia (18/1484, 1.21%) (χ^2^ = 551.735, *p* < 0.001). After controlling for HAMA and HAMD scores, the MetS rate was 58.32 times higher in FEDND patients with hyperglycemia than in FEDND patients without hyperglycemia (B = 4.066, Wald statistic = 221.221, *p* < 0.001, OR = 58.32, 95% CI = 34.13–99.65).

### Comparison of clinical characteristics and biochemical parameters between MetS and non-MetS in FEDND patients with and without hyperglycemia

As shown in Table 1, univariate analysis of FEDND patients without hyperglycemia revealed significant differences in demographic and clinical characteristics between MetS and non-MetS, including age, age of onset, marital status, severe anxiety, and suicide attempts (all *p* < 0.05). Patients with MetS were older, had a later age of onset, and were predominantly married than those without MetS (all *p* < 0.05). And patients with MetS had higher HAMD, HAMA, and PANSS positive subscale scores, as well as higher TSH, TgAb, and TC levels than non-MetS patients (all *p* < 0.05). Among FEDND patients without hyperglycemia, the suicide attempt rate was much higher in MetS patients (12/18, 66.67%) than in non-MetS patients (251/1466, 17.12%). In addition, the prevalence of SHypo was higher in FEDND patients with MetS (83.33%, 15/18) than in non-MetS patients (51.64%, 757/1466).

**Table 1:** Socio-demographics and clinical characteristics of FEDND patients without and with MetS

Variable	MDD without HG		*U/X* ^*2*^ */t*	*p*	MDD with HG		*U/X* ^*2*^ */t*	*p*
	Without MetS (N = 1466)	With MetS (N = 18)			Without MetS (N = 133)	With MetS (N = 101)		
Age, years	34.61 ± 12.34	44.94 ± 11.31	19309.000	**0.001**	32.50 ± 12.38	39.95 ± 11.98	9023.000	**< 0.001**
Age of onset, years	34.40 ± 12.23	44.56 ± 11.45	19267.500	**0.001**	32.40 ± 12.25	39.07 ± 11.90	9006.000	**< 0.001**
Marital status			5.049	**0.033**			5.942	**0.017**
Sigle, *n* (%)	438 (29.88%)	1 (5.60%)			44 (33.10%)	19 (18.80%)		
Married, *n* (%)	1028 (70.10%)	17 (94.40%)			89 (66.90%)	82 (81.20%)		
Suicide attempt *n* (%)	251 (17.12%)	12 (66.67%)	29.935	**< 0.001**	44 (33.10%)	39 (38.60%)	0.767	0.410
Severe Anxiety *n* (%)	149 (10.16%)	6 (33.33%)	10.205	**0.008**	23 (17.30%)	26 (25.70%)	2.476	0.144
HAMD	30.10 ± 2.91	32.28 ± 2.97	18533.000	**0.003**	31.44 ± 3.12	31.28 ± 2.51	6391.500	0.523
HAMA	20.58 ± 3.39	23.44 ± 3.73	19161.500	**0.001**	21.77 ± 3.55	22.27 ± 3.76	7210.500	0.333
Psychotic positive score	8.58 ± 4.08	11.28 ± 6.51	17633.500	**0.001**	9.84 ± 5.20	10.99 ± 6.11	7080.000	0.421
TSH, mIU/L	4.71 ± 2.35	6.69 ± 2.65	-3.548	**< 0.001**	6.99 ± 2.67	7.44 ± 2.83	-1.238	0.217
FT3, pmol/L	4.92 ± 0.73	4.86 ± 0.70	12229.000	0.593	4.82 ± 0.68	4.86 ± 0.64	-0.532	0.595
FT4, pmol/L	16.68 ± 3.12	16.09 ± 2.43	11585.000	0.373	17.03 ± 3.15	16.67 ± 2.74	0.924	0.357
TPOAb, IU/L	62.79 ± 136.82	91.83 ± 123.07	15345.000	0.234	97.34 ± 187.79	173.32 ± 353.00	6487.500	0.655
TgAb, IU/L	83.71 ± 226.56	271.39 ± 459.10	19320.000	**0.001**	108.97 ± 239.91	124.36 ± 321.59	7143.000	0.406
TC, mmol/L	5.17 ± 1.08	5.78 ± 1.14	17688.000	**0.013**	5.68 ± 1.17	5.90 ± 1.10	-1.457	0.147
LDL-C, mmol/L	2.94 ± 0.84	3.28 ± 0.94	15475.000	0.207	3.27 ± 1.01	3.28 ± 0.83	-0.116	0.908

Note: HG: Hyperglycemia; HAMD: Hamilton Rating Scale for Depression; HAMA: Hamilton Anxiety Rating Scale; TC: total cholesterol; LDL-C: low density lipoprotein cholesterol; TSH: thyroid stimulating hormone; FT3: free triiodothyronine; FT4: free thyroxine; TgAb: antithyroglobulin; TPOAb: thyroid peroxidases antibody

However, among FEDND patients with hyperglycemia, univariate analysis showed significant differences in demographic and clinical characteristics between MetS and non-MetS, including age, age of onset, and marital status (*p* < 0.05). Patients with MetS were older, had a later age of onset, and were predominantly married than those without MetS, consistent with FEDND patients without hyperglycemia. We did not observe any other significant differences in clinical variables and biomarkers (all *p* > 0.05).

### Risk factors for MetS in FEDND patients with and without hyperglycemia

**Fig. 1:**
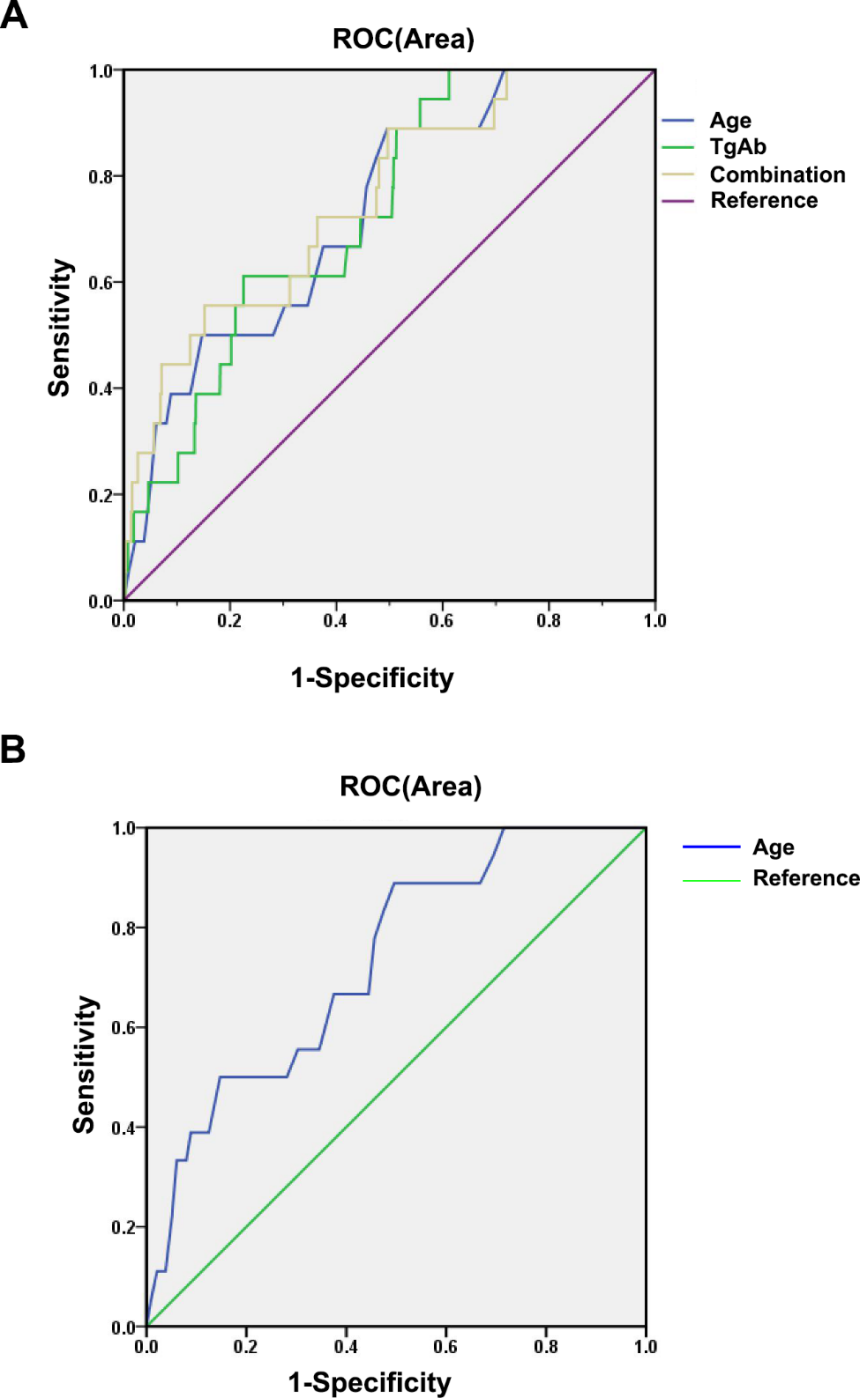
The discriminatory capacity of related factors for distinguishing between patients with and without MetS in FEDND with/without hyperglycemia. **(A)** The area under the curve of age, TgAb, and combination of two factors between patients with and without MetS in FEDND patients without hyperglycemia were 0.732, 0.732, and 0.754, respectively. **(B)** The area under the curve of age between patients with and without MetS in FEDND patients with hyperglycemia was 0.732

We then focused on MetS risk factors in FEDND patients with and without hyperglycemia. Variables that differed significantly in univariate analysis and those that were clinically related to MetS were included in logistic regression (condition: Wald) to detect risk factors for MetS in patients with FEDND with and without hyperglycemia. As shown in Table 2, in patients with FEDND without hyperglycemia, the risk factors for MetS were age (B = 0.066, *p* = 0.002, OR = 1.068, 95% CI = 1.024–1.115), suicide attempts (B = 2.167, *p* < 0.001, OR = 8.734, 95% CI = 3.208–23.773) and TgAb levels (B = 0.001, *p* = 0.035, OR = 1.001, 95% CI = 1.000-1.002). The AUCROC showed a value of 0.732 for both age and TgAb. The combination of age and TgAb had a higher AUC value of 0.754, which could differentiate MetS from non-MetS (*p* < 0.0001. 95% CI = 0.643–0.865) (Fig. 1A).

**Table 2:** Risk factors for MetS in FEDND patients without and with hyperglycemia

	Without HG					With HG				
	B	Wald statistic	p	OR	95%CI	B	Wald statistic	p	OR	95%CI
Age	0.066	9.173	0.002	1.068	1.024–1.115	0.048	18.757	0.000	1.050	1.027–1.073
Suicide attempt	2.167	17.992	< 0.001	8.734	3.208–23.773					
TgAb	0.001	4.447	0.035	1.001	1.000-1.002					

Note: HG: Hyperglycemia; TgAb: anti-thyroglobulin;

However, only age (B = 0.048, *p* < 0.001, OR = 1.050, 95% CI = 1.027–1.073) was an independent risk factor for MetS in FEDND patients with hyperglycemia. Moreover, the AUCROC showed a value of 0.732 for age (*p* = 0.001, 95% CI = 0.626–0.837) (Fig. 1B).

## Discussion

This is the first study investigating MetS and its associated risk factors in FEDND patients with and without hyperglycemia. In this study, we found that (1) the prevalence of MetS was much higher in FEDND patients with hyperglycemia (43.16%) than in FEDND patients without hyperglycemia (1.21%); (2) age was an independent risk factor for MetS in FEDND patients with hyperglycemia; (3) age, suicide attempts, and TgAb were independent risk factors for MetS in FEDND patients without hyperglycemia; and (4) in patients with FEDND without hyperglycemia, the combination of age and TgAb distinguished between MetS and non-MetS (AUC was 0.754).

### Factors associated with MetS in FEDND patients with hyperglycemia

Studies on the prevalence and risk factors of MetS in patients with MDD are scarce. To date, only a few cross-sectional studies have reported the prevalence of MetS in patients with MDD. Even less has been conducted on the prevalence and factors associated with MetS in FEDND patients with and without hyperglycemia. A meta-analysis by Vancampfort *et al*. showed that patients with MDD had a higher prevalence of MetS (30.5%) and a higher risk of hyperglycemia and hypertriglyceridemia compared to healthy people of the same age and sex [39]. Several studies have shown that antidepressants increase the risk of MetS [29, 40, 41]. The prevalence of MetS in patients with FEDND in this study (6.93%) was much lower than in other studies, probably due to differences in race, geography, and diagnosis. In addition, and most importantly, the participants included in this study were patients with first-episode MDD who had not received any antipsychotic medication, which may also explain the low prevalence of MetS. All studies have shown that MDD patients with MetS have higher FBG and TG levels than those without MetS [7, 39, 42, 43]. Our study also showed that the prevalence of MetS was more than 35 times higher in FEDND patients with hyperglycemia than in those without hyperglycemia. This indicates that the prevalence and risk factors for MetS are significantly different in patients with and without hyperglycemia. Although patients with MDD are 1.2–2.6 times more likely to develop T2D than those without MDD, most patients with MDD still have blood glucose levels within the normal range, especially those with first-episode depression (1484/1718) [19, 44]. Investigating the differences in MetS risk factors between MDD patients with and without hyperglycemia is more clinically relevant for the treatment of MDD and prevention of T2D and CVD.

### Factors associated with MetS in FEDND patients without hyperglycemia

The current study found that the risk factors for MetS were different in FEDND patients with and without hyperglycemia. In the hyperglycemic state, only age, age of onset, and marital status were associated with MetS, while the severity of depression and anxiety were no longer key factors in the development of MetS. Numerous studies have confirmed that both T2D and CVD are age-related physiological disorders, and their risk increases with age [45, 46]. Our results showed that in FEDND patients with and without hyperglycemia, the age and age of onset were significantly higher in patients with MetS than in those without MetS, suggesting that the older the patients, the higher the age of onset, the higher the risk of developing MetS in FEDND patients with or without hyperglycemia. In addition, our study showed that marital status was associated with MetS in FEDND patients with or without hyperglycemia. An association between marital status and the presence of CVD and T2D and their associated adverse outcomes has been found [47, 48]. Those who remain married are much less likely to develop T2D and CVD than those who are divorced or separated. Dissatisfaction with marriage and marital quality strongly influence T2D, CVD, and MDD. The association between marital status and CVD/T2D/MDD outcomes may be influenced by psychosocial, economic, and other acute stressors; however, the underlying processes are unknown.

However, in patients with FEDND without hyperglycemia, the factors associated with MetS were more complex. In FEDND patients without hyperglycemia, suicide attempts, severe anxiety, HAMA, HDMA, and PANSS positive subscale scores were associated with MetS in addition to age, age of onset, and marital status. Several studies have shown that the severity of MDD patients is positively associated with MetS [49]. Our findings further established that the severity of depression was associated with MetS only in FEDND patients without hyperglycemia, but not in FEDND patients with hyperglycemia. In addition, among FEDND patients without hyperglycemia, TSH, TgAb, and TC levels were higher in patients with MetS than in those without MetS. One previous study found that elevated TSH levels and SHypo were positively correlated with glucose disorders and MetS [50]. Our study confirmed that TSH levels were not correlated with MetS in FEDND patients with hyperglycemia, but significantly correlated with MetS in FEDND patients without hyperglycemia. TgAb and TPOAb are clinical markers of autoimmune thyroid disease (AITD) and are the underlying cause of SHypo and hypothyroidism, leading to thyroid dysfunction [51, 52]. Among FEDND patients without hyperglycemia, the prevalence of SHypo was higher in patients with MetS (83.33%, 15/18) than in those without MetS (51.64%, 757/1466). This result further supports that SHypo increases the risk of MetS in MDD patients without hyperglycemia [53]. In the present study, among FEDND patients without hyperglycemia, TgAb levels were significantly higher in MetS patients than in non-MetS patients. These results suggest a strong correlation between SHypo and MetS, which is consistent with the results of other studies [53].

### Combination of age and thyroid function distinguished MetS from non-MetS in FEDND patients without hyperglycemia

Analyses of logistic regression showed that age was the only independent risk factor for MetS in FEDND patients with hyperglycemia. Age is considered as a potential influencing factor for MetS [54]. Andrea *et al*. reported that the prevalence of MetS increased with age in German MDD patients under 60 years of age [43]. Consistent with our findings, older MDD patients had an increased risk of MetS. On the other hand, in FEDND patients without hyperglycemia, logistic regression analysis showed that age, suicide attempts, and TgAb were independent risk factors for MetS. Moreover, among FEDND patients without hyperglycemia, suicide attempt rate was much higher in patients with MetS (12/18, 66.67%) than in non-MetS patients (251/1466, 17.12%). In a study of patients with psychotic major depression, DBP was found to be an independent risk factor for suicide attempts [55]. This may suggest an association between suicide attempts and MetS. Elevated TgAb, a biomarker of AITD, predicts the development of thyroid dysfunction, especially SHypo. Our study further found that in FEDND patients without hyperglycemia, elevated TgAb was a risk factor for MetS. However, in FEDND patients with hyperglycemia, factors related to thyroid function are no longer independent risk factors for MetS. In FEDND patients with hyperglycemia, we could distinguish MetS from non-MetS only by age, while in FEDND patients without hyperglycemia, the combination of age and thyroid function helped us to distinguish MetS from non-MetS better, suggesting that in FEDND patients without hyperglycemia, the combination of age and TgAb slightly improved our ability to distinguish between MetS and non-MetS.

### Limitations

As a case-control study, the present study had some limitations. (1) We could not determine the causal relationship between hyperglycemia and MetS in MDD patients. Follow-up prospective studies could be conducted to investigate whether there are causal relationship between MetS and hyperglycemia in FEDND patients. (2) The participants in this study were all Han Chinese, so it is uncertain whether our findings are generalized to other ethnic and clinical groups. (3) For marital status, we did not specify whether singles were divorced or unmarried or had partners, and whether married people were satisfied with their marriage.

## Conclusions

In conclusion, the incidence of MetS was much higher in FEDND patients with hyperglycemia than in FEDND patients without hyperglycemia. Age was an independent risk factor for MetS in FEDND patients with hyperglycemia. In contrast, age, TgAb, and suicide attempt were independent risk factors for MetS in FEDND patients without hyperglycemia. The combination of age and TgAb distinguished MetS from non-MetS in patients with FEDND. Controlling blood glucose levels and improving thyroid function are essential to reduce the incidence of MetS in patients with MDD, and are particularly important in older patients.

## Data Availability

Data analyzed in this study is subject to the following licenses/restrictions: All data in the current study are stored in affiliates of the PI. And is available from the corresponding author upon reasonable request and completion of the data user agreement. For access to these datasets, please get in touch with Professor Xiangyang Zhang, zhangxy@psych.ac.cn.
